# Supplementary material for: Are genomic language models all you need? Exploring genomic language models on protein downstream tasks
Source: Bioinformatics. 2024 Aug 30;40(9):btae529. doi: 10.1093/bioinformatics/btae529 (PMC11399231; doi:10.1093/bioinformatics/btae529)
Supplement: btae529_Supplementary_Data [file btae529_supplementary_data.pdf]

Supplementary Tables

Dataset	Task Type	Experiment Type	# Train Samples	# Validation Samples	# Test Samples	Mean Sequence Length (bp)
Fluorescence	Regression	Single Protein Mutagenesis	21464	5366	27217	714
Beta-Lactamase Activity (Unique)	Regression	Single Protein Mutagenesis	3457	865	1080	858
Beta-Lactamase Activity (Complete)	Regression	Single Protein Mutagenesis	11252	2814	1080	858
Stability	Regression	Multi-Protein	53700	2512	12851	135
Melting Point	Regression	Multi-Protein	9432	1064	1648	1176
Secondary Structure Prediction	Per AA Classification	Multi-Protein	6224	1556	334	724

Table 1: Overview of the tasks in the curated CDS dataset. Samples in each task's dataset contain protein sequences paired with CDS sequences. Total sampled over all 3 test sets is provided for SSP.

	Melting Point (R^2)	Stability (ρ)	Fluorescence (ρ)	Beta- Lactamase (ρ)		SSP (Acc)		
Train Dataset	All	All	Unique	Unique	Complete	All		
Test Dataset	All	All		All	All	CASP12	CB513	TS115
NT500M	0.82 ± 0.01	0.69 ± 0.02	0.68 ± 0.00	0.76 ± 0.04	0.81 ± 0.01	0.51 ± 0.00	0.52 ± 0.01	0.52 ± 0.00
DNABert2	0.81 ± 0.01	0.52 ± 0.09	0.67 ± 0.00	0.61 ± 0.02	0.64 ± 0.03	NA	NA	NA
ESM2	0.69 ± 0.02	0.77 ± 0.04	0.68 ± 0.00	0.89 ± 0.00	0.89 ± 0.01	0.63 ± 0.01	0.75 ± 0.00	0.76 ± 0.00
ESM1b	0.69 ± 0.02	0.79 ± 0.02	0.68 ± 0.00	0.87 ± 0.01	0.88 ± 0.01	0.62 ± 0.01	0.72 ± 0.00	0.71 ± 0.00
Joint (ESM + NT)	0.82 ± 0.01	0.81 ± 0.03	0.68 ± 0.01	0.89 ± 0.00	0.89 ± 0.00	NA	NA	NA

Table 2: Evaluation results of Nucleotide Transformer v2 (500M), DNABERT2, ESM2 (650M), ESM1-b (650M), and joint ESM-NTv2 models on the test sets of the different tasks. The metrics used to measure performance in each task were chosen to match previous benchmarks and include Spearman correlation ρ, R^2, and accuracy, with a higher value indicating better performance for all metrics. We note that protein models evaluated on *Complete* splits see identical proteins with different labels, but we still performed the evaluation for completeness. DNABERT2 was not evaluated on SSP since its BPE tokenization prevents residue-level predictions.

Sampling Strategy	Melting Point True	Melting Point Per	Melting Point Uni	Fluorescence True	Fluorescence Per	Fluorescence Uni	Fluorescence Complete
NT500M	0.82 ± 0.01	0.80 ± 0.01	0.64 ± 0.01	0.68 ± 0.00	0.67 ± 0.01	0.48 ± 0.08	0.68 ± 0.00
DNABert2	0.81 ± 0.01	0.78 ± 0.01	0.55 ± 0.01	0.67 ± 0.00	0.61 ± 0.00	0.13 ± 0.02	0.67 ± 0.00
NT50M (6mer)	0.81 ± 0.01	0.78 ± 0.01	0.59 ± 0.01	0.68 ± 0.00	0.63 ± 0.00	0.28 ± 0.01	0.67 ± 0.00
NT50M (3mer, 600B tokens)	0.82 ± 0.01	0.82 ± 0.00	0.63 ± 0.01	0.68 ± 0.00	0.66 ± 0.00	0.41 ± 0.06	0.68 ± 0.00
NT50M (3mer, 300B tokens)	0.81 ± 0.01	0.82 ± 0.01	0.63 ± 0.01	0.68 ± 0.00	0.66 ± 0.00	0.33 ± 0.01	0.68 ± 0.00
ESM2	0.69 ± 0.02	NA	NA	0.68 ± 0.00	NA	NA	0.68 ± 0.00
ESM1b	0.69 ± 0.02	NA	NA	0.68 ± 0.00	NA	NA	0.68 ± 0.00

Sampling Strategy	Stability True	Stability Per	Stability Uni	Beta-Lactamase True	Beta-Lactamase Per	Beta-Lactamase Uni	Beta-Lactamase Complete
NT500M	0.69 ± 0.02	0.69 ± 0.04	0.63 ± 0.03	0.76 ± 0.04	0.09 ± 0.06	0.06 ± 0.03	0.81 ± 0.01
DNABert2	0.52 ± 0.09	0.54 ± 0.03	0.44 ± 0.04	0.61 ± 0.02	0.00 ± 0.04	0.01 ± 0.02	0.64 ± 0.03
NT50M (6mer)	0.63 ± 0.04	0.62 ± 0.03	0.51 ± 0.02	0.66 ± 0.01	0.01 ± 0.02	0.02 ± 0.03	0.72 ± 0.01
NT50M (3mer, 600B tokens)	0.58 ± 0.07	0.64 ± 0.04	0.63 ± 0.03	0.72 ± 0.01	0.05 ± 0.03	0.06 ± 0.05	0.78 ± 0.01
NT50M (3mer, 300B tokens)	0.59 ± 0.03	0.59 ± 0.05	0.60 ± 0.04	0.74 ± 0.01	0.05 ± 0.05	0.04 ± 0.04	0.79 ± 0.01
ESM2	0.77 ± 0.04	NA	NA	0.89 ± 0.00	NA	NA	0.89 ± 0.01
ESM1b	0.79 ± 0.02	NA	NA	0.87 ± 0.01	NA	NA	0.88 ± 0.01

Sampling Strategy	CASP12 True	CASP12 Per	CASP12 Uni	CB513 True	CB513 Per	CB513 Uni	TS115 True	TS115 Per	TS115 Uni
NT500M	0.51 ± 0.00	0.62 ± 0.00	0.58 ± 0.00	0.52 ± 0.01	0.62 ± 0.00	0.57 ± 0.00	0.52 ± 0.00	0.61 ± 0.00	0.57 ± 0.00
DNABert2	NA	NA	NA	NA	NA	NA	NA	NA	NA
NT50M (6mer)	0.48 ± 0.00	0.56 ± 0.00	0.52 ± 0.00	0.48 ± 0.00	0.56 ± 0.00	0.52 ± 0.00	0.48 ± 0.00	0.56 ± 0.00	0.52 ± 0.00
NT50M (3mer, 600B tokens)	0.51 ± 0.01	0.59 ± 0.00	0.55 ± 0.00	0.52 ± 0.00	0.59 ± 0.00	0.55 ± 0.00	0.52 ± 0.00	0.59 ± 0.00	0.55 ± 0.00
NT50M (3mer, 300B tokens)	0.51 ± 0.00	0.58 ± 0.00	0.54 ± 0.00	0.51 ± 0.01	0.58 ± 0.00	0.55 ± 0.00	0.51 ± 0.01	0.58 ± 0.00	0.55 ± 0.00
ESM2	0.63 ± 0.01	NA	NA	0.75 ± 0.00	NA	NA	0.76 ± 0.00	NA	NA
ESM1b	0.62 ± 0.01	NA	NA	0.72 ± 0.00	NA	NA	0.71 ± 0.00	NA	NA

Table 3: Performance of the different models across all tasks using different CDS datasets based on different codon sampling strategies: True CDS (True), Permuted (Per), Unique (Uni) and Complete.

Dataset	Fine-tuning Steps
Melting Point	20000
Beta-Lactamase	200000
Secondary Structure	20000
Stability	50000
Fluorescence	100000

Table 4: Maximum number of fine-tuning steps per dataset. Models were early stopped based on the validation set performance using the task's respective metric with a patience of 30 steps. However, when early stopping was not invoked, we set a maximum number of training steps per task based on the amount of data.

Appended	Nothing	GC	Species	GC + Species
ESM2	0.69 ± 0.02	0.71 ± 0.03	0.79 ± 0.01	0.79 ± 0.01
NTv2	0.82 ± 0.01	0.82 ± 0.01	0.83 ± 0.01	0.83 ± 0.01

Table 5: Performance of NTv2 and ESM models on the melting point prediction task when trained solely on protein/CDS sequences (Nothing) or with additional information about GC-content, species of origin, or both. The metric used is the R2.

Model	CASP12	CB513	TS115
NTv2	0.65 ± 0.00	0.72 ± 0.00	0.74 ± 0.00
ESM2	0.73 ± 0.01	0.87 ± 0.00	0.86 ± 0.00
ESM1b	0.71 ± 0.01	0.84 ± 0.00	0.84 ± 0.00
PSIPRED	0.76	0.82	0.83
S4Pred	0.68	0.73	0.75

Table 6: Performance of NTv2, ESM and classic models on three-way classification task for the secondary structure prediction datasets. The metric reported is accuracy.

Helices			Strands		Coils		
3_{10} helix	α-helix	π-helix	β-sheet	β-bridge	turn	bend	coil (other)

Table 7: Breakdown of Secondary Structure Types

Supplementary Figures

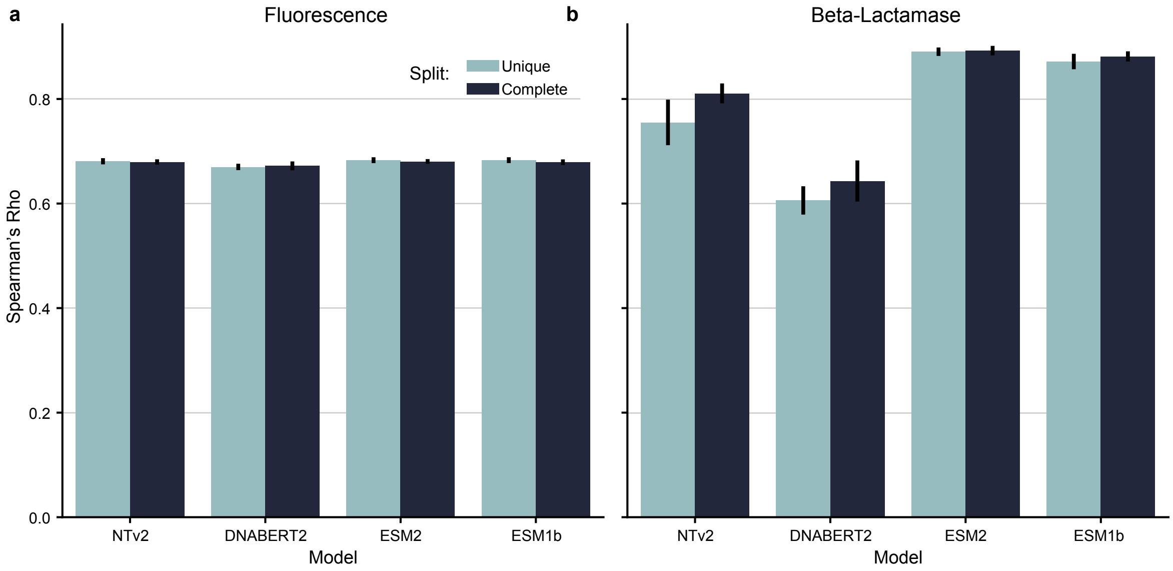

Supplementary Figure 1: **Training on Synonymous Codon Improves gLM Beta-Lactamase Performance.** **a)** Model performances is nearly identical between Fluorescence Complete and Unique set. This is because, as described in Methods A.2.1, there are hardly any degenerate sequences. **b)** Performance of models on Beta-Lactamase Complete vs Unique datasets, evaluated on the same test set. GLMs trained on the complete dataset outperform those trained on the Unique Set. Unlike Fluroescence there are many more degenerate sequences in the Complete Set. There is no observed effect for pLMs.

.

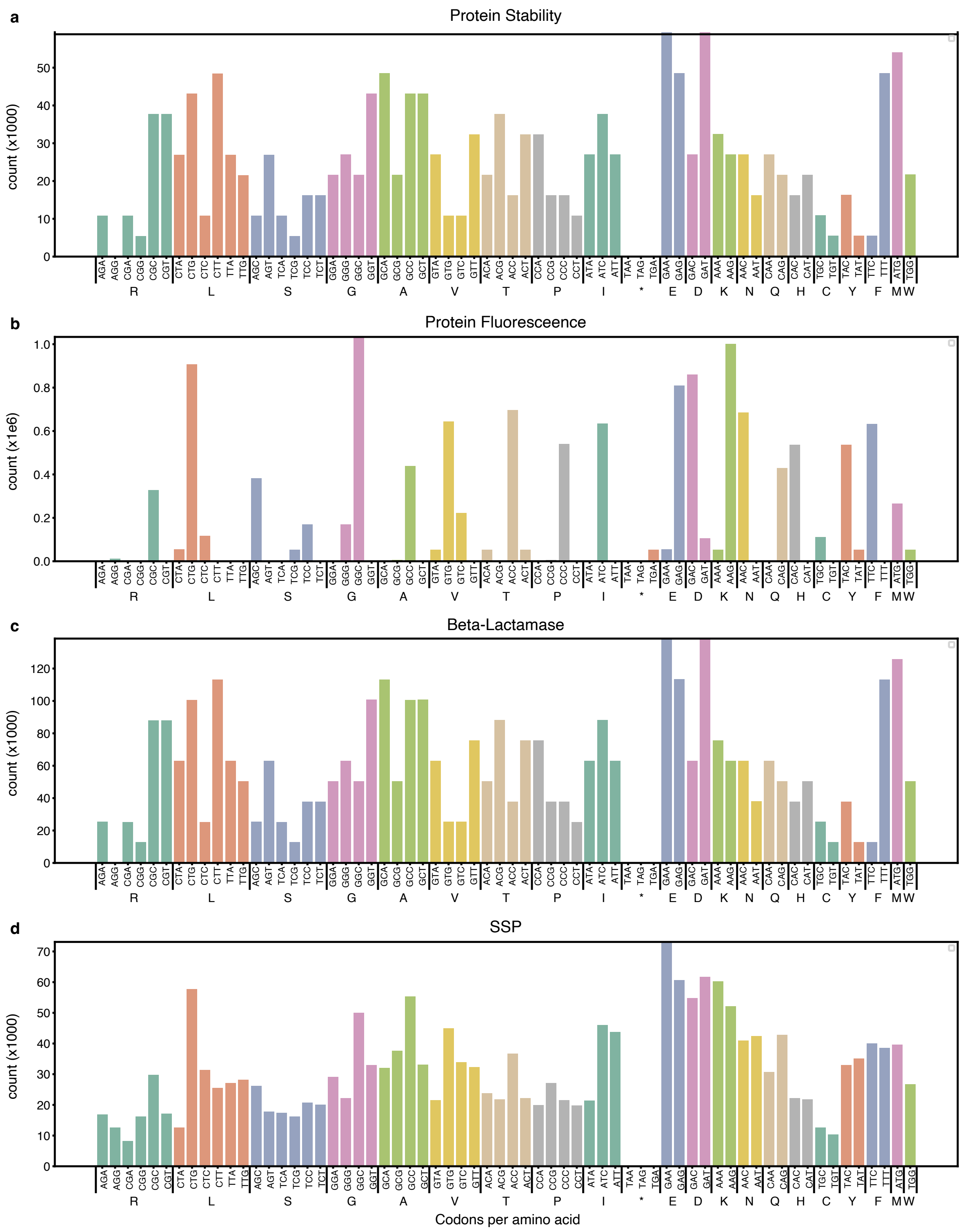

Supplementary Figure 2: Codon frequency for the proteins present in each dataset

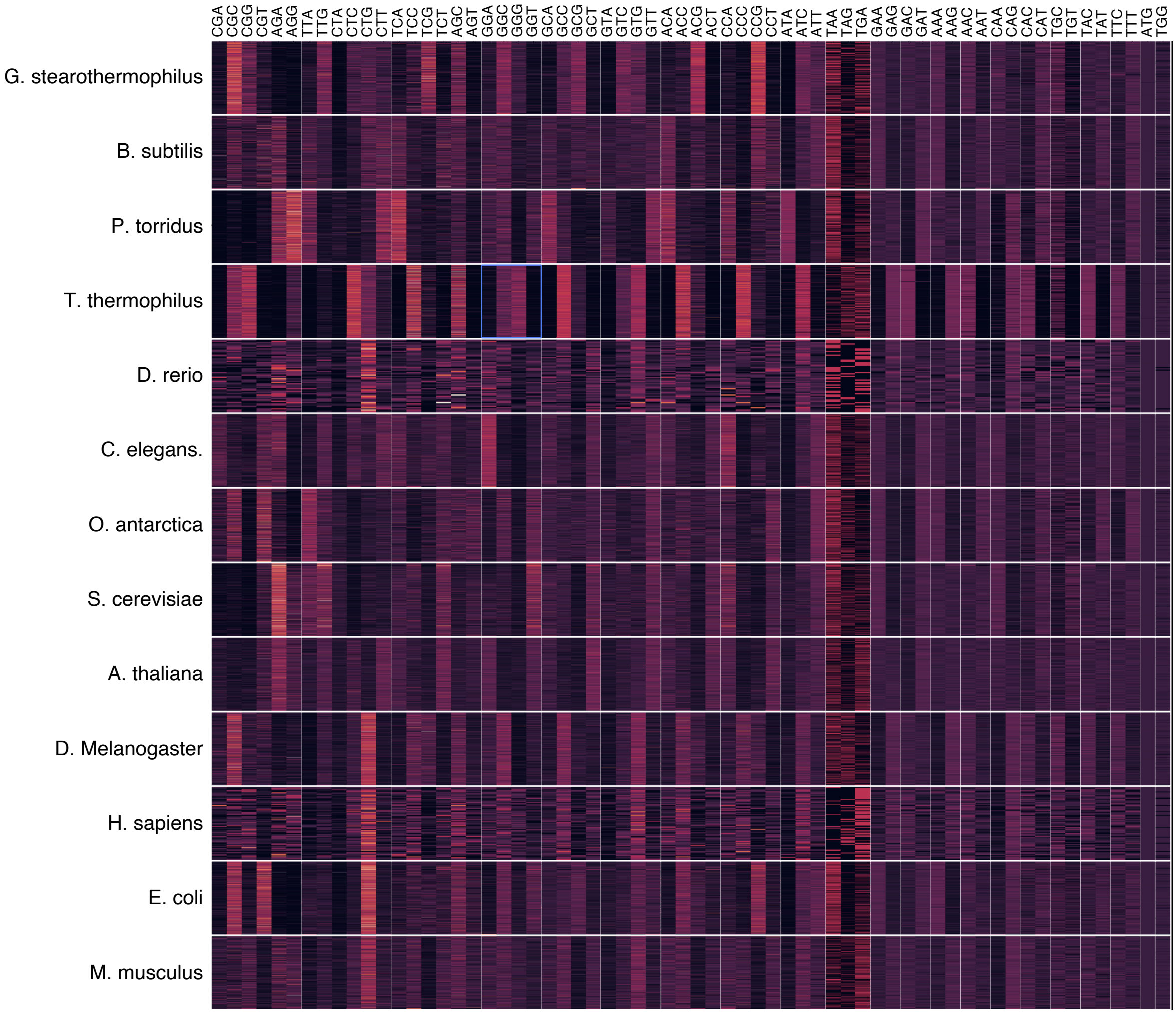

Supplementary Figure 3: **Relative synonymous codon usage per species.** This figure shows the relative synonymous codon usage (RSCU) of each sequence in the melting point task, partitioned by species. RCSU is defined as the observed codon frequency over the expected codon frequency assuming synonymous codons are equally likely. In the figure, columns are amino acids and sub-columns are codons. Rows are species, and sub-rows are individual sequences with that species. Thus, the color at some sub-row, sub-col location denotes the RCSU of some codon in a a sequence of a particular species, where bright colors are closer to 1. The figure reveals many species have strong and identifiable synonymous codon preferences.

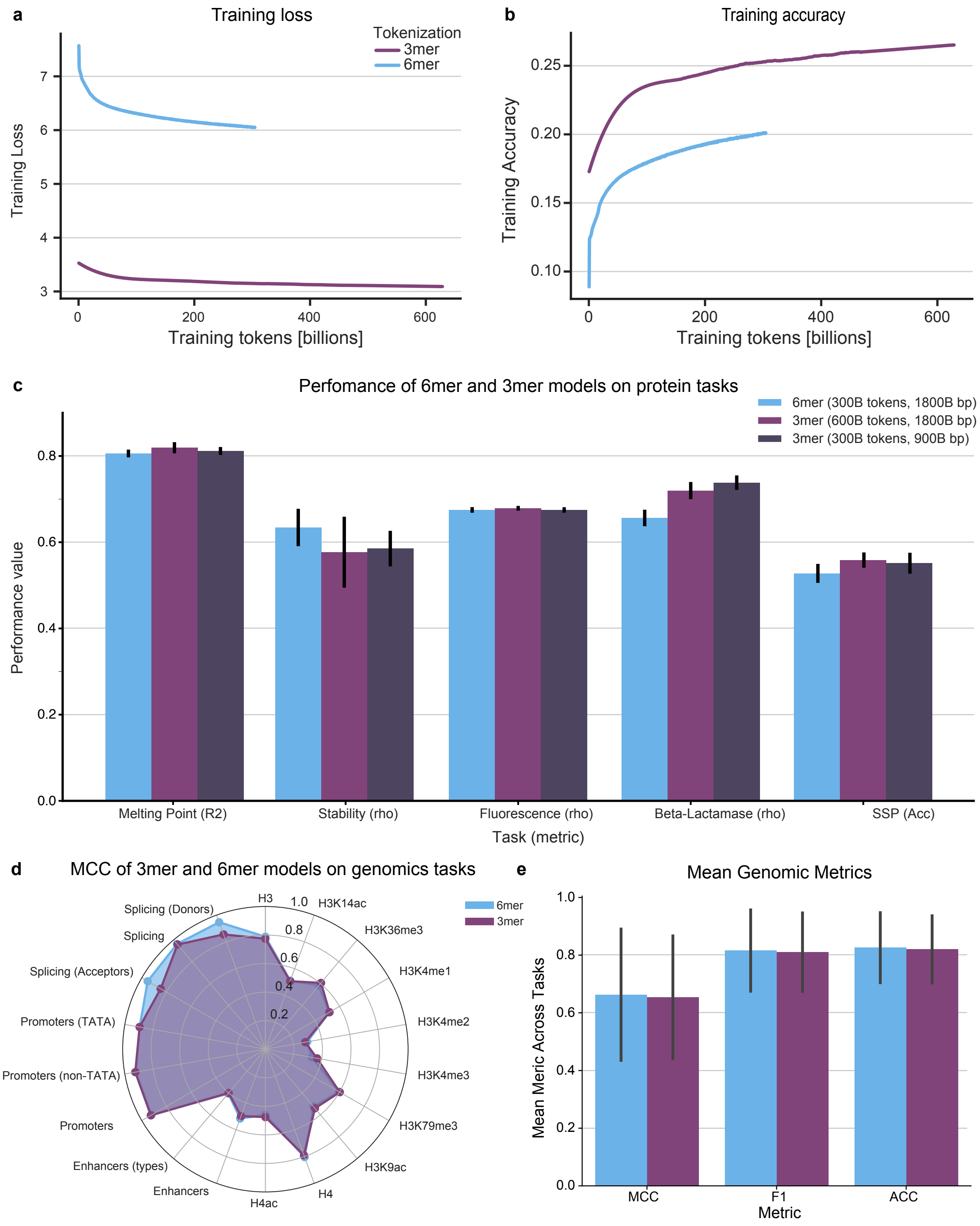

Supplementary Figure 4: **Improved performance of 3mer over 6mer NTv2 on protein tasks. a-b)** Training curves for the pre-training of the 3mer and 6mer NTv2 models. Training loss **(a)** and accuracy **(b)** are shown in function of the number of training tokens (billions). **c)** Performance of the 6mer and two checkpoints of the 3mer model per task. The metric used for each task is mentioned in the x-axis labels. **d)** Matthew's correlation coefficient (MCC) for 3mer (300B tokens) vs 6mer tokenized models across 18 genomics tasks. **e)** Mean MCC, F1-score and Accuracy across all genomics tasks for 3mer (300B tokens) and 6mer models.

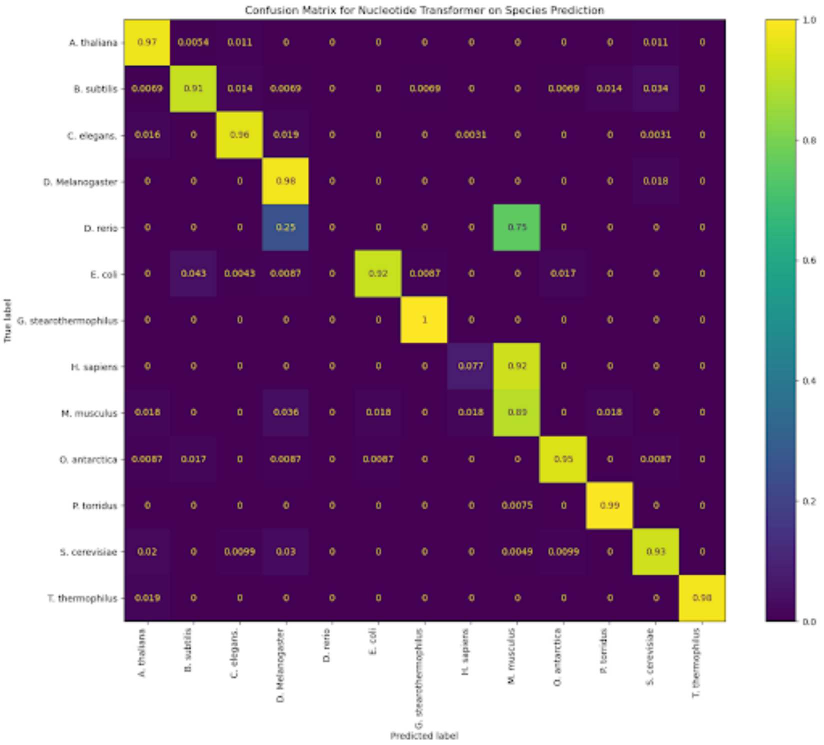

Supplementary Figure 5: **Confusion matrix for NTv2 model on species prediction.**

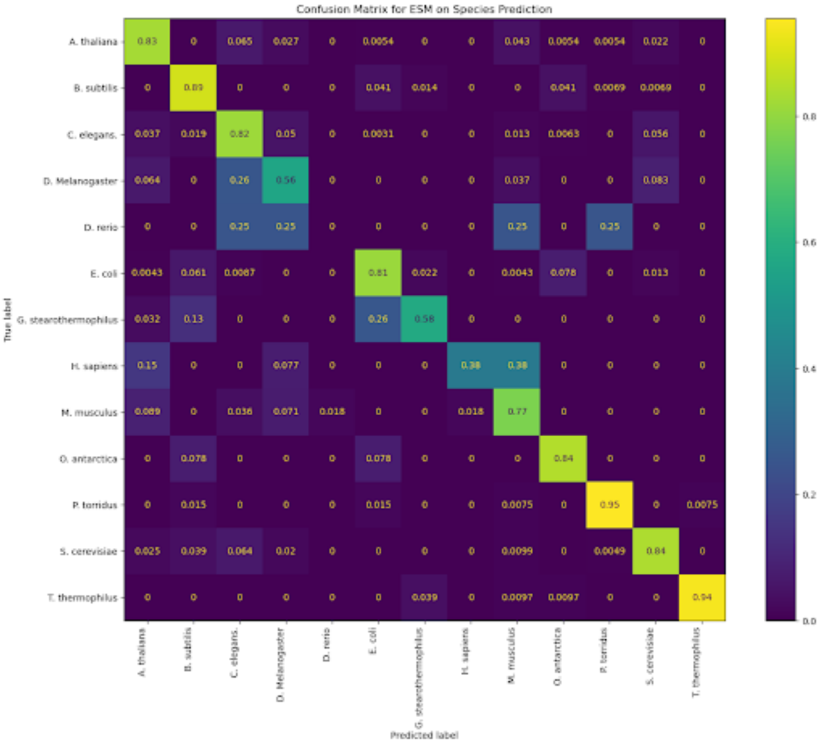

Supplementary Figure 6: **Confusion matrix for ESM model on species prediction.**

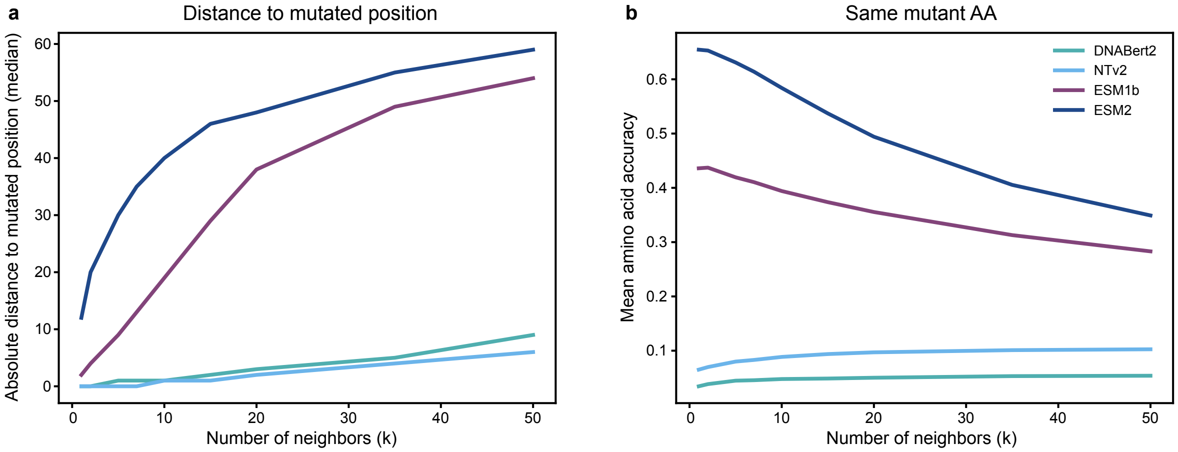

Supplementary Figure 7: **gLM embeddings capture the mutated position while pLM embeddings capture the mutant amino acid identity. a)** Absolute distance to median mutated position of neighbouring sequences in function of the number of neighbours of each query sequence. This relationship is shown for the embeddings of the four different models. **b)** Mean accuracy for the prediction of the mutated amino acid identity of the query sequence based on the mutated amino acid of the neighbouring sequences, in function of the number of neighbours of each query sequence. This relationship is shown for the embeddings of the four different models.
